# Prevalence of metabolic syndrome in human immunodeficiency virus - infected patients from the South-West region of Cameroon, using the adult treatment panel III criteria

**DOI:** 10.1186/1758-5996-6-92

**Published:** 2014-08-25

**Authors:** Herbert Afegenwi Mbunkah, Henry Dilonga Meriki, Anthony Tufon Kukwah, Omarine Nfor, Theresa Nkuo-Akenji

**Affiliations:** Department of Microbiology and Parasitology, University of Buea, P.O. Box 63, Buea, Cameroon; Faculty of Science Clinical Diagnostic Laboratory, University of Buea, P.O. Box 63, Buea, Cameroon; Buea Regional Hospital, Buea, Cameroon; Department of Medical Laboratory Science, University of Bamenda, Bamenda, Cameroon

**Keywords:** Metabolic syndrome, Antiretroviral therapy, HIV, HAART

## Abstract

**Background:**

Several studies have reported that the metabolic syndrome (MS) is more common in subjects with HIV infection than in HIV-negative individuals. HIV infection and the use of Highly Active Antiretroviral Therapy (HAART) have been shown to predispose HIV-infected persons to MS. In this study, we report the prevalence of MS in Cameroonian HIV-infected subjects receiving different combinations of HAART as well as HIV patients who have never received antiretroviral drugs.

**Methods:**

In this cross-sectional study, 173 treated and untreated HIV-infected out-patients (aged 18–70 years) managed at the Buea and Limbe Regional Hospitals and 50 seronegative individuals (controls) were recruited after obtaining their consent. Ethical approval for this study was obtained from the National Ethics Committee of Cameroon. Metabolic syndrome prevalence was examined using the U.S. National Cholesterol Education Program Adult Treatment Panel III (ATPIII) criteria. Data was analyzed using SPSS® (Statistical Package for the Social Sciences, SPSS Inc., Chicago, IL, USA) version 16. Statistical significance was set at p < 0.05.

**Results and discussion:**

The prevalence of MS among the HIV patients was 15.6% (27/173) and 8% (4/50) among the controls and the difference was significant (p = 0.022). MS was more prevalent in HIV-infected patients on HAART than in ART-naive patients and seronegative individuals. Overall, the prevalence of MS was significantly higher (p = 0.003) in females (28/153; 18.3%) than in males (3/70; 4.3%). The patients on first-line drugs demonstrated the highest MS prevalence (15/62; 24.2%) followed by the ART-naïve group of patients (7/61; 11.5%) and the lowest prevalence was among the patients on protease inhibitors (5/50; 10%). Patients on the drug combination Lamivudine/Stavudine/Nevirapine had the highest prevalence of MS (50%).

**Conclusions:**

In this study, HAART but not HIV disease plays a significant role in the development of MS. The metabolic complications as a result of treatment with HAART may predispose HIV patients to developing cardiovascular diseases and diabetes, in spite of improvements in morbidity and mortality conferred by immune reconstitution as a result of HAART treatment.

## Background

The use of antiretroviral therapy (ART) especially the highly active antiretroviral therapy (HAART) has led to a significant reduction in AIDS related morbidity and mortality [1]. Although ART has positively modified the natural history of HIV, long-term toxicity is becoming recognized. In addition a variety of metabolic abnormalities including dyslipidemia, fat redistribution, high blood pressure, and insulin resistance have frequently been associated with ART, particularly when it contains protease inhibitors [2].

The National Cholesterol Education Program’s Adult Treatment Panel (ATP) III report identified the metabolic syndrome (MS) as a multiplex risk factor for cardiovascular disease and defined it as the occurrence of three or more of the following abnormalities: hypertriglyceridemia, low high-density lipoprotein (HDL) cholesterol, hypertension, abdominal obesity, and high serum glucose [3]. Several studies have reported that the metabolic syndrome is more common in subjects with HIV infection than in HIV-negative individuals [4–6]. The components of metabolic syndrome have been recognized in patients infected with HIV [7–9].

There is limited information on MS prevalence in HIV-infected patients receiving HAART worldwide, especially in the present study site (Cameroon). A Spanish study reported a prevalence of 17% by the ATP III criteria [10]. Most patients who come down with MS are at a greater risk of developing coronary heart disease (CHD) and diabetes. In this study, we report the prevalence of MS in HIV-infected subjects receiving different combinations of HAART, HIV patients who have never received antiretroviral drugs, as well as seronegative individuals (controls).

## Research design and methods

This was a cross-sectional study carried out on HIV-infected out-patients managed at the Buea and Limbe Regional Hospitals of the South West Region of Cameroon over a period of 9 months, from November 2010 through July 2011. These hospitals have the major HIV treatment centres in the Region. Buea (coordinates: 4°10′0 N 9°14′0E) is the capital of the South West Region of Cameroon located on the eastern slopes of Mount Cameroon. Results of the 2005 census revealed that Buea has a population of 150,000 people. Limbe (coordinates: 4°01′ N 9°13′ E) with a population of 84,223 is a natural resource coastal city. The out-patients came from Buea, Limbe and other surrounding villages (Muea, Tole, Ekona, Mutengene, Bolifamba, Ombe, Bova and Idenau). The ethical clearance for this study was one issued by the National Ethics Committee in Cameroon for an on-going related and larger study on HIV/AIDS co-infections.

All participants were evaluated by trained physicians after giving their informed consent. Blood samples were collected into dry vacutainer tubes after a 12-hour overnight fast and analysed at the Clinical Diagnostic Laboratory of the University of Buea. A total of 241 participants (aged 18–70 years) were enrolled having fulfilled the inclusion criteria of the study (HIV positive people; untreated and those receiving treatment for at least 1 month who after giving their consent, voluntarily accepted to take part). Pregnant women and persons who did not satisfy the inclusion criteria were excluded. Six participants were on anti-diabetic drugs and twelve on antihypertensive drugs and were therefore excluded from the study. Of the 223 qualified participants, 62 were HIV-infected patients on first-line drug treatment, 50 were HIV-infected patients on second-line drug treatment, 61 were untreated (ART-naive) HIV-infected patients and a last group of 50 was made up of HIV negative individuals (controls). First-line drug treatment was a combination of 2 Nucleotide Reverse Transcriptase Inhibitors (NRTIs) + a Non-Nucleoside Reverse Transcriptase Inhibitor (NNRTI) while second-line drug treatment was a combination of 2 NRTIs + 2 Protease Inhibitors (P.Is).

A rapid test to screen for the presence of anti-HIV antibodies was performed using the rapid test kit, Determine™ HIV-1/2 (Abbot Laboratories, Japan). Confirmation of all results was done using the ImmunoComb® II HIV-1/2 Bispot kit. Weight, height and waist circumference were measured by standard methods and the body mass index (BMI) calculated. After the patient had rested for 10 minutes seated in a quiet room, blood pressure (BP) was measured in the left arm with the elbow flexed at heart level by a physician using an electronic BP machine (Airial® Bp2200). Two readings were obtained, and the average of the systolic and diastolic blood pressure readings was used. Total serum cholesterol and serum triglycerides were determined using enzymatic-colorimetric methods in a Mindray® BA-88 Biochemistry analyzer using the Cholesterol- HB006 and Triglycerides- HB021 Kits (Cypress Diagnostics Ltd.) respectively. HDL-cholesterol was measured using the phosphotungstic precipitation method with phosphotungstic acid and magnesium ions (HDL-Cholesterol- HB007 Kit- Cypress Diagnostics Ltd.). LDL-Cholesterol was calculated using the Friedewald formula [11]. Fasting blood glucose was determined using a glucometer (Clever Chek® TD-4222) following the manufacturer’s instructions. All participants mounted on a body fat analyzer (Tanita® Bodyfat Analyzer) to obtain a complete body fat analysis. CD4 counts of the HIV patients were obtained using the Partec® CyFlow Counter (Partec Gmbh) according to the manufacturer’s instructions.

In the ATP III report of 2001, individuals with three or more of the following criteria are defined as having the metabolic syndrome: waist circumference >102 cm in men and >88 cm in women; triglycerides ≥150 mg/dL (1.69 mmol/L); HDL cholesterol <40 mg/dL (1.04 mmol/L) in men and <50 mg/dL (1.29 mmol/L) in women; blood pressure ≥130/85 mmHg; and fasting plasma glucose ≥110 mg/dL (6.1 mmol/L).

All statistical calculations were done using the computer program SPSS® (Statistical Package for the Social Sciences, SPSS Inc., Chicago, IL, USA) version 17. Charts were produced using Microsoft® Excel 2007. Comparison of group means of all parameters was performed using the ANOVA test. Categorical data and the prevalence of MS in HIV-infected patients were compared using the Pearson’s Chi-Square test. A *p*-value < 0.05 was considered statistically significant.

## Results

A total of 241 participants were enrolled into the study but 18 were disqualified because they were either on anti-diabetics or anti-hypertensive drugs. Of the remaining 223, there were 70 (31.4%) males and 153 (68.6%) females. A majority of the naïve-ART group of patients were in the primary clinical stage of infection while those on second-line treatment (protease inhibitor-containing HAART) group were at clinical stage 1 of infection. This latter group also had the highest percentage of patients (24%) at clinical stage 2. The mean CD4 count was highest among the naïve-ART group of patients, followed by the first-line treatment group of patients. No participant was on lipid lowering drugs. The details of the demographic, anthropometric, HIV status and other characteristics of the 223 participants are shown in Table 1.

**Figure 1:**
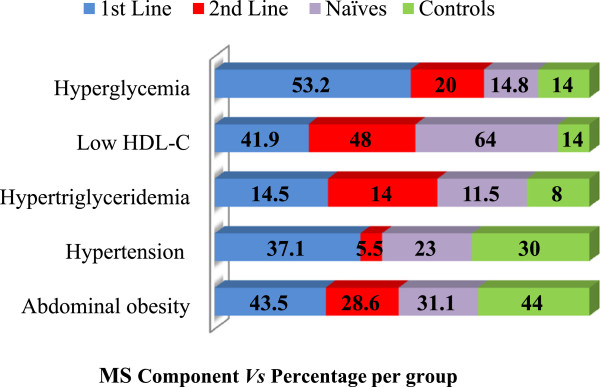
**Prevalence of the components of MS.**

The prevalence of the components of MS was also evaluated. The highest to the lowest prevalent component was low HDL-C (43%), abdominal obesity (36.8%), hyperglycemia (26.5%), hypertension (24.7%) and hypertriglyceridemia (12.1%). The frequency distribution of the occurrence of these components is shown on Figure 1.

**Table 1:** **Demographic, anthropometric, HIV status and other characteristics of the 223 participants**

Characteristics	1 ^st^Line drugs	2 ^nd^Line drugs	Naïve-ART	Controls
	(n = 62)	(n = 50)	(n = 61)	(n = 50)
	n (%)	n (%)	n (%)	n (%)
HIV status	Positive	Positive	Positive	Negative
Gender				
Female	48 (77.4)	31 (62)	44 (72.1)	30 (60)
Male	14 (22.6)	19 (38)	17 (27.9)	20 (40)
Mean Age ± SD	41.1 ± 11.2	38.7 ± 11.3	36.3 ± 11.8	47.3 ± 13.7
Mean CD4 count ± SD (cells/μL)	382 ± 173.2	215.1 ± 119	399 ± 212.7	/
Mean BMI (kg/m^2^) ± SD	24.6 ± 4.2	23 ± 3.5	23.6 ± 3.8	28.6 ± 6
Smokers	2 (3.2)	0 (0)	3 (4.9)	1 (2)
Alcohol consumers	4 (6.5)	0 (0)	10 (16.4)	4 (8)
Aerobic exercise	20 (32.3)	7 (14)	18 (29.5)	12 (24)
Fat mass (Kg): mean (SD)	18.7 (9.7)	15.3 (9.2)	17.9 (9.5)	26.7 (17.9)
Lean Body Mass (Kg): mean (SD)	47.1 (8.9)	46.4 (9.6)	45.7 (11.3)	49.3 (9.9)
Total body water (Kg): mean (SD)	34.3 (6.7)	33.9 (7.0)	34.1 (8.5)	36.0 (7.2)
Clinical staging				
Primary	51 (82.3)	2 (4)	54 (88.5)	/
Stage 1	11 (17.7)	36 (72)	5 (8.2)	/
Stage 2	0 (0)	12 (24)	2 (3.3)	/
WHO BMI classification				
Underweight	5 (8.1)	7 (14)	10 (16.4)	3 (6)
Normal	33 (53.2)	29 (58)	31 (50.8)	14 (28)
Overweight	18 (29)	10 (20)	17 (27.9)	13 (26)
Obese class 1	5 (8.1)	2 (4)	3 (4.9)	13 (26)
Obese class 2	1 (1.6)	0 (0)	0 (0)	4 (8)
Obese class 3	0 (0)	0 (0)	0 (0)	3 (6)

The overall prevalence of MS in this study (Table 2) was 13.9% with significant differences in the prevalence among groups (p = 0.022). The prevalence of MS among the HIV-infected patients was 15.6% (27/173) and 8% (4/50) among the controls. The MS prevalence among the ART-naive and control groups was compared and no statistically significant difference (p = 0.542) was found. A similar comparison was done between all patients on HAART and the ART-naive group. A statistically significant difference (p = 0.020) was found. In the study population, the prevalence of MS was significantly higher (p = 0.003) in females (28/153; 18.3%) than in males (3/70; 4.3%). In those on first-line drug treatment, the prevalence of MS was 29.2% and 7.1% for females and males respectively. Similarly, when second-line treatment was considered, the prevalence was 10% for females and 0% for males.

**Table 2:** **Prevalence of metabolic syndrome among groups**

Treatment	Prevalence	P-value
1^st^ Line drug treatment	15/62 (24.2%)	
2^nd^ Line drug treatment	5/50 (10%)	0.022*
Naïve	7/61 (11.5%)	
Controls	4/50 (8%)	χ^2^ = 9.6
**Overall prevalence**	31/223 (13.9%)	

*Statistically significant.

The relationship between drug combination and MS was also investigated. MS was most prevalent in patients receiving the drug combination Lamivudine/Stavudine/Nevirapine (Table 3). Within the HIV patients, the metabolic syndrome was significantly associated with age, BMI, waist circumference, fasting blood sugar, triglycerides and to a lesser extent, HDL cholesterol and sex (Table 4).

**Table 3:** **Prevalence of MS with respect to drug combination**

Drug combination (n)	MS (%)
Lamivudine/Zidovudine/Nevirapine^†^ (35)	6 (17.1%)
Lamivudine/Zidovudine/Efavirens^†^ (15)	5 (33.3%)
Lamivudine/Stavudine/Nevirapine^†^ (6)	3 (50%)
Lamivudine/Stavudine/Efavirens^†^ (6)	1 (16.7%)
Lopinavir/Ritonavir/ Lamivudine/Zidovudine ^♣^ (17)	2 (11.8%)
Lopinavir/Ritonavir/Tenofovir/Lamivudine ^♣^ (14)	0 (0%)
Lopinavir/Ritonavir/Tenofovir/Emtricitabine ^♣^ (19)	3 (15.8%)

^†^ = First-line drug combination ^♣^ = Second-line drug combination n = number.

**Table 4:** **Association of age, sex, HIV disease stage, lipodystrophy and HAART with the metabolic syndrome**

	Patients with MS	Patients without MS	p-value
**Number (%)**	27 (15.6)	146 (84.4)	
**Mean age in yrs. (SD)**	44.9 (11.2)	37.5 (11.5)	0.001*
**Sex (%)**			
Male	2 (7.4)	37 (25.3)	0.040*
Female	25 (92.6)	109 (74.7)	
**Mean BMI in kg/m** ^**2**^ **(SD)**	25.2 (3.4)	23.7 (4.0)	0.002*
**HIV disease stage (%)**			
Primary	20 (74.1)	86 (58.9)	0.123
Stage 1	3 (11.1)	44 (30.1)	
Stage 2	4 (14.8)	16 (11)	
**Mean CD4 cell count in cells/μL (SD)**	412.4 (173.6)	348.2 (202.1)	0.248
**Mean waist circumference in cm (SD)**	94.37 (6.9)	85.11 (7.7)	0.001*
**Mean HDL-C in mg/dL (SD)**	37.7 (11.9)	48.6 (17.5)	0.043*
**Mean FBS in mg/dL (SD)**	111.6 (13.0)	91.4 (20.4)	0.001*
**Mean triglyceride in mg/dL (SD)**	125.5 (73.0)	83.7 (40.4)	0.014*
**Antiretroviral therapy exposure**			
Naïve-ART (%)	7 (25.9)	54 (37)	0.065
Never on protease inhibitor (%)	15 (55.6)	47 (32.2)	
Currently protease inhibitor (%)	5 (18.5)	45 (30.8)	
**Mean years of HIV infection duration (SD)**	3.6 (1.1)	2.7 (0.8)	0.242
**Mean years of HAART duration (SD)**	2.4 (0.7)	1.9 (0.3)	0.148

*Statistically significant.

The lipid profile differed among the groups. A statistically significant difference existed in the mean concentration of total cholesterol (TC) between the first-line and second-line patients (p = 0.027) and also between the first-line and the naïve (untreated) patients (p = 0.01). The mean concentration of HDL-C was significantly higher among the HAART-treated patients than in the untreated patients (p = 0.021). The mean concentrations of TC and HDL-C were significantly higher in the controls than in the treated cases (p = 0.040 and p = 0.032 respectively). Dyslipidemia was prevalent among the first-line group of patients (14.5%) followed by the second-line group of patients (14%) while the naïve-ART patients had a prevalence of 11%. In the controls, the prevalence was 12%. Overall, the difference in these prevalence rates was not significant (p = 0.792).

## Discussion, conclusions and recommendations

The widespread use of highly active antiretroviral therapy (HAART) has resulted in a dramatic decrease in the morbidity and mortality of patients infected with HIV. Unfortunately, HAART is increasingly associated with the emergence of adverse metabolic events. There is evidence of MS following infection with HIV and the administration of HAART in our study population.

Risk factors of MS such as increased age, BMI, hypertension and hyperglycemia were higher among the HIV patients diagnosed with MS than in patients without MS. The MS prevalence among the ART-naive and control groups was compared and no statistically significant difference (p = 0.542) was found, indicating that there isn’t a role for HIV infection on developing the MS. A similar comparison was done between all patients on HAART and the ART-naive group. It was found out that HAART plays a significant role in the development of MS (p = 0.020). The prevalence of MS was not affected by HIV disease stage. Patients with MS had a higher mean CD4 cell count. The type or duration of antiretroviral therapy was not an independent risk factor for MS. These findings are similar to those of a prospective, cross-sectional study of the risk factors associated with MS and cardiovascular disease (CVD) among HIV patients conducted in the US in 2005. It was shown that HIV-infected patients with MS were older, had a high CD4 cell count and body mass index, compared to patients without MS and the type or duration of ART was not an independent risk factor for MS [12]. However, the study conducted in Barcelona (2005) by Jericŏ and colleagues revealed that patients with MS presented with lower CD4 cell counts but higher ages and BMI than patients without MS.

Our study demonstrated that low HDL-C (43%), abdominal obesity (36.8%), hyperglycemia (26.5%), hypertension (24.7%) and hypertriglyceridemia (12.1%) were the prevalent MS components. In a cross-sectional study in Barcelona using 710 HIV-infected patients, Jericŏ *et al*. [10] demonstrated that hypertriglyceridemia (95%) was the most frequent trait of MS, followed by low HDL cholesterol (71.1%), high blood pressure (67.8%), abdominal obesity (47.1%), and high blood glucose levels (46.3%). Another study with 477 HIV-infected adults in the US revealed that the most common metabolic abnormalities were low HDL (54%) and high triglycerides (47%) and the least common was high blood glucose (4%). The prevalence of high blood pressure and abdominal obesity were 33% and 25% respectively [13]. In our study, the prevalence of MS components was dissimilar among the first-line, second-line and ART-naïve patients. This is contrary to findings in 2007 by Bonfanti *et al*. [14] where the prevalence of MS components was similar in treated and never-treated HIV-infected patients. A larger sample size is needed to confirm the preliminary findings in the present study.

The prevalence of MS among the HIV patients was 15.6% and 8% among the uninfected group (controls). This is in line with several studies which have reported that the metabolic syndrome is more common in subjects with HIV infection than in HIV-negative individuals [4–6]. In a cross-sectional study with 710 HIV-infected patients in Barcelona, Spain, the prevalence of MS was 17% and only stavudine and lopinavir/ritonavir were independently associated with the metabolic syndrome [10]. In this study, the prevalence of MS was significantly higher in females than in males. In previous studies, women were also reported to be more frequently diagnosed with MS than men [15, 16].

MS was more prevalent among patients on first-line treatment than those on second-line drugs (P.Is). Our finding contradicts those from other studies reporting that MS is usually most prevalent among HIV patients on protease inhibitor-based HAART. In 2007, an international cross-sectional study of a well-characterized cohort of 788 HIV-infected adults revealed an 18% prevalence of the metabolic syndrome and protease inhibitor use was associated with a significantly higher prevalence of MS [15]. Jacobson and colleagues in 2006 demonstrated in their cohort study that lopinavir/ritonavir users had a higher risk of developing the metabolic syndrome [13]. Our study also demonstrated that patients on the drug combination Lamivudine/Stavudine/Nevirapine had the highest prevalence of the MS (50%). The drug stavudine has been noted to be strongly associated with MS [10, 15, 17].

In conclusion, MS is more prevalent in HIV-infected patients on HAART (especially those taking the Lamivudine/Stavudine/Nevirapine drug combination) than in ART-naive patients and seronegative individuals. Although numerous studies have implicated P.Is as an important risk factor for cardiovascular disease, the use of P.Is was not found to be an independent risk factor for MS in the present study. Our study indicates that there isn’t a role for HIV infection on developing the MS but the use of HAART plays a significant role in the development of MS. The metabolic complications as a result of treatment with HAART left HIV patients at a risk of developing CVD and diabetes in spite of improvements in morbidity and mortality conferred by immune reconstitution as a result of HAART treatment. Although the substantial benefits of combination ART clearly outweigh the increase in cardiovascular risk associated with this therapy, it must be borne in mind that with progressive aging of the HIV-infected population and the expected long-term use of combination ART, it is important to prevent an increased incidence of MS in this population.

For future studies, we recommend:

- ➢ The use of larger sample sizes and the recruitment of patients with longer ART exposure periods.
- ➢ That data should be obtained on pre-existing risk factors for metabolic syndrome prior to becoming HIV-positive since this could alter results on MS prevalence.
- ➢ The inclusion of patients in the late stages of infection.

## Authors’ information

**MHA**: MSc. Microbiology; Current PhD student, Department of Microbiology and Parasitology, University of Buea Cameroon.

**MHD**: PhD Microbiology; Instructor, Department of Microbiology and Parasitology, University of Buea Cameroon; Laboratory Scientist, Buea Regional Hospital Cameroon.

**KAT**: MSc. Microbiology; Current PhD student, Department of Microbiology and Parasitology, University of Buea Cameroon; Laboratory Scientist, Buea Regional Hospital Cameroon.

**NO**: MSc. Medical Microbiology and Parasitology; Current PhD student; Assistant Lecturer, Department of Medical Laboratory Science, University of Bamenda Cameroon.

**N-AT**: Professor of Parasitology; Deputy Vice-Chancellor i/c Internal Control and Evaluation, University of Buea Cameroon.

## Abbreviations

AIDS: Acquired immunodeficiency syndrome
ART: Antiretroviral therapy
BMI: Body mass index
BP: Blood pressure
CD4: Cluster of differentiation 4
CVD: Cardiovascular disease
HAART: Highly active antiretroviral therapy
HDL-C: High density lipoprotein cholesterol
HIV: Human immunodeficiency virus
mg/dL: Milligrams per deciliter
mmHg: Millimeters of mercury
mmol/L: Millimoles per litre
MS: Metabolic syndrome
NNRTI: Non-nucleoside reverse transcriptase inhibitor
NRTI: Nucleoside reverse transcriptase inhibitor
NtRTI: Nucleotide reverse transcriptase inhibitor
PIs: Protease inhibitors
SD: Standard deviation
SPSS: Statistical package for social sciences
χ^2^: Chi-squared.
